# The association between soy consumption and metabolic syndrome in Chinese adults: a cross-sectional study

**DOI:** 10.3389/fnut.2025.1637413

**Published:** 2025-08-15

**Authors:** Khemayanto Hidayat, Yan-Hui Huang, Xiao-Yan Qian, Xiao-Fang Chen, Lu-Gang Yu, Hui Zhou, Li-Qiang Qin

**Affiliations:** ^1^Department of Nutrition and Food Hygiene, School of Public Health, Soochow University, Suzhou, China; ^2^Suzhou Industrial Park Centers for Disease Control and Prevention, Suzhou, China

**Keywords:** soy, metabolic syndrome, blood pressure, lipid, glucose, obesity

## Abstract

**Background:**

Evidence on the association between soy consumption and metabolic syndrome (MetS) remains limited and inconclusive. This study aimed to investigate the relationship between soy intake and the prevalence of MetS and its components in a Chinese population.

**Methods:**

A cross-sectional analysis was conducted among 5,107 adults residing in Suzhou Industrial Park, Suzhou, China. Dietary intake was assessed using an interviewer-administered food frequency questionnaire. MetS was defined according to the Joint Interim Statement (JIS) criteria. Logistic regression models were used to estimate odds ratios (ORs) and 95% confidence intervals (CIs) for MetS and its components in relation to soy consumption, adjusting for potential confounders.

**Results:**

Each 25 g/day increase in soy consumption was associated with lower odds of MetS (OR 0.95, 95% CI 0.92–0.98), elevated waist circumference (OR 0.97, 95% CI 0.94–0.99), elevated triglycerides (OR 0.94, 95% CI 0.91–0.96), reduced HDL-cholesterol (OR 0.95, 95% CI 0.92–0.97), and elevated blood pressure (OR 0.97, 95% CI 0.94–0.99), but not elevated fasting blood glucose. These associations were more pronounced and consistent among women. Menopausal status did not significantly modify the associations.

**Conclusion:**

Higher soy consumption was associated with a lower prevalence of MetS and most of its components, particularly among women. These findings highlight the potential role of soy foods in metabolic health and warrant prospective studies to clarify causal relationships, assess individual soy food types, and explore sex- and menopause-specific effects.

## Introduction

Metabolic syndrome (MetS) refers to a cluster of cardiometabolic risk factors, including elevated fasting plasma glucose, elevated blood pressure, elevated triglycerides, reduced high-density lipoprotein (HDL) cholesterol, and increased waist circumference. These factors are collectively associated with increased risks of type 2 diabetes mellitus (T2DM) and cardiovascular diseases (1). The global prevalence of MetS has risen over time and represents a growing public health concern in many countries (2–4). In China, the prevalence increased from 8.8% between 1991 and 1995 to 29.3% between 2011 and 2015, with a more pronounced rise observed in women (from 7.9 to 30.7%) compared to men (from 9.4 to 27.2%) (4). These trends underscore the importance of identifying modifiable risk factors to inform effective prevention strategies.

Dietary factors are believed to contribute to both the development and potential prevention of MetS. Plant-based dietary patterns have been proposed as a protective factor against MetS and related conditions, although supporting evidence continues to evolve (5).

Soy foods are a prominent component of traditional diets in East Asian countries, including China, Korea, and Japan. Soy is low in saturated fat and provides complete protein (including all nine essential amino acids), unsaturated fatty acids, dietary fiber, isoflavones, and various micronutrients (6). Among these components, soy protein and isoflavones have been studied for their potential health effects (6). Clinical trials suggest that short-term soy supplementation, primarily through isolated soy protein or isoflavones, may improve certain MetS components (6), including lipid profiles (7, 8), blood pressure (9, 10), glucose metabolism (11), and waist circumference (12). However, these findings may not directly translate to habitual soy food consumption in free-living populations.

Several observational studies, including prospective cohort (13) and cross-sectional studies (14–18), have examined associations between soy protein or isoflavone intake and MetS in East Asian populations. The findings remain inconsistent: some studies report inverse associations (13, 18), while others report null (14, 15, 17) or positive associations (16). Additionally, sex may modify the associations with cardiometabolic outcomes, with some studies suggesting more favorable associations in women (13, 14), while findings in men are less consistent or even unfavorable (13, 14, 19, 20).

Given these inconsistencies, further investigation is needed to clarify the associations between habitual soy food consumption and MetS, particularly in Chinese populations. This study aims to examine these associations using health survey data from residents of Suzhou Industrial Park (21, 22).

## Materials and methods

### Study design, setting, and population

This study employed a cross-sectional design. Participants were recruited from four local communities in the Suzhou Industrial Park (Suzhou City, Jiangsu Province, China) as part of a community-based health survey conducted between July 2013 and November 2014 (21, 22). All community residents aged 18 years or older were invited to participate through public announcements and outreach at community health service centers. A total of 7,866 individuals— ~65% of the invited population—provided written informed consent and completed the baseline survey.

As this study was a secondary analysis of existing survey data, no formal sample size calculation was conducted *a priori*. Instead, we included all available participants with complete data on soy consumption and all five MetS components. No additional exclusion criteria—such as preexisting medical conditions or pregnancy—were applied in order to preserve the generalizability of the findings. All interviews, physical examinations, biological sample collections, and clinical measurements were conducted by trained personnel using standardized protocols. The study was conducted in accordance with the Declaration of Helsinki, and the protocol was approved by the Ethics Committee of Soochow University (approval number: ECSU-2010-002).

### Blood samples and measurements

Fasting blood samples were collected via venipuncture following a 10–12 h overnight fast. Plasma concentrations of glucose, total cholesterol, triglycerides, HDL cholesterol, and low-density lipoprotein (LDL) cholesterol were measured using an automatic analyzer (Olympus AU640, Kobe, Japan). Systolic and diastolic blood pressure were measured in a seated position using a calibrated mercury sphygmomanometer (Shanghai Zhangdong Med-Tech Ltd., Shanghai, China). Anthropometric measurements were obtained by trained personnel following standardized procedures. Height and waist circumference were measured to the nearest 0.1 cm, and weight to the nearest 0.1 kg. Body mass index (BMI) was calculated as weight in kilograms divided by the square of height in meters (kg/m^2^).

### Dietary assessment

Dietary intake was assessed using a structured, interviewer-administered food frequency questionnaire (FFQ) designed to capture habitual intake over the past year. Participants were asked to report the frequency and typical portion size of selected food groups, including soy, fruits, vegetables, nuts, salted vegetables, red meat, poultry, fish, and milk. Soy consumption was assessed using a single item labeled “soybeans and soy products.” Although individual soy foods were not recorded separately, the questionnaire was administered by trained interviewers who clarified that this category included commonly consumed soy-based foods in Chinese diets, such as tofu, soy milk, soybean paste, and soybeans. Participants selected one of five frequency categories (never, yearly, monthly, weekly, or daily) and reported usual portion size using standard household units (斤 [jin], 两 [liang], or ml). Daily consumption of soy and other food groups (g/day) was calculated by multiplying reported portion size by frequency and converting the result to grams using standard food conversion factors. Although the FFQ was not designed for detailed nutrient profiling, it enabled the quantification of major food groups.

### Other covariates

Demographic and behavioral variables were collected through a structured interviewer-administered questionnaire. Covariates included age, sex, menopausal status, educational level, alcohol consumption, smoking status, physical activity, sleep duration, television viewing duration, and use of medications for diabetes, dyslipidemia, or hypertension. Menopausal status was determined based on self-reported cessation of menstruation, with women classified as postmenopausal if they reported having undergone natural menopause or surgery (i.e., oophorectomy); Otherwise, they were considered premenopausal.

### Definition of metabolic syndrome

MetS was defined according to the Joint Interim Statement (JIS) criteria established by several international health organizations (1). The presence of MetS was determined by meeting at least three of the following five criteria:

1. Elevated waist circumference (Asian-specific cut-offs): ≥90 cm for men or ≥80 cm for women;
2. Elevated triglyceride: ≥150 mg/dl (1.7 mmol/L), or current use of lipid-lowering medication;
3. Reduced HDL cholesterol: < 40 mg/dl (1.0 mmol/L) in men or < 50 mg/dl (1.3 mmol/L) in women, or current use of lipid-lowering medication;
4. Elevated blood pressure: systolic blood pressure ≥130 mmHg or diastolic blood pressure ≥85 mmHg, or current use of antihypertensive medication;
5. Elevated fasting glucose: ≥100 mg/dl (5.6 mmol/L), or current use of antidiabetic medication (oral or insulin).

The JIS criteria were chosen for the present study because they are widely used in international and regional epidemiological research and incorporate Asian-specific cut-offs suitable for Chinese adults. This definition enhances comparability with global studies, has been widely adopted in national surveillance efforts (17, 23, 24), and has demonstrated higher sensitivity for identifying MetS in various Chinese populations compared to the Chinese Diabetes Society (CDS) criteria (25, 26).

### Data analysis

Participants were categorized into quartiles based on their estimated average daily soy food consumption (g/day). Differences in participant characteristics across quartiles of soy consumption were assessed using the chi-squared (χ^2^) test for categorical variables and one-way analysis of variance (ANOVA) for continuous variables. A multivariable logistic regression model was used to estimate odds ratios (ORs) and 95% confidence intervals (CIs) for MetS and each of its components across quartiles of soy consumption, with the lowest quartile as the reference group. Additionally, soy consumption was modeled as a continuous variable to estimate the ORs (95% CIs) associated with each 25 g/day increase in soy consumption. This cut-off was selected based on the Chinese Dietary Guidelines (2022), which recommend a combined daily intake of 25–35 g of soybeans and nuts for adults (27). Given prior evidence suggesting that sex and menopausal status may modify the association between soy consumption and cardiometabolic outcomes (13, 14, 19, 20), analyses were stratified accordingly. All regression models were adjusted for age, sex (not included as covariates in sex- and menopause-stratified models), educational level, smoking status, alcohol consumption, physical activity, sleep duration, television viewing duration, BMI, and the consumption of red meat, poultry, fish, nuts, salted vegetables, vegetables, and fruits. Model fit was evaluated using Nagelkerke *R*^2^, a pseudo-*R*^2^ statistic commonly used in logistic regression to quantify the proportion of variance in the outcome explained by the exposure and covariates. All statistical analyses were performed using SPSS version 20.0 (SPSS Inc., Chicago, IL, USA). All *P*-values were two-sided, and a *P*-value < 0.05 was considered statistically significant.

## Results

Of the 7,866 participants, 624 were excluded due to incomplete information on soy consumption, and 2,135 due to missing data on any MetS component, resulting in a final analytical sample of 5,107 participants. The characteristics of study participants are summarized in Table 1. The mean age was 54 ± 9.60 years, and 55% were women. Regarding educational attainment, 54% had less than a high school education. Additionally, 70% reported not consuming alcohol, and 80% reported not smoking tobacco. Compared with those in the lowest quartile of soy consumption, individuals in higher quartiles tended to be younger, reported longer sleep duration, and spent less time watching television. Alcohol consumption differed across quartiles of soy consumption. Higher soy consumption was also accompanied by lower consumption of red meat and higher consumption of fruits, vegetables, poultry, fish, dairy, nuts, and salted vegetables. Anthropometric and biochemical parameters—including body weight, BMI, waist circumference, systolic and diastolic blood pressure, triglyceride, LDL cholesterol, and fasting blood glucose—tended to be highest among participants in the lowest quartile of soy consumption. In contrast, HDL cholesterol levels were lowest in this group.

**Table 1:** Characteristics of the study participants according to soy foods consumption.

**Variable**	**Q1 (*n* =1,294)**	**Q2 (*n* =1,333)**	**Q3 (*n* =1,260)**	**Q4 (*n* =1,220)**	P^*^
Soy in g/day, mean (SD)	9.66 ± 5.95	24.70 ± 3.99	41.85 ± 11.32	137.45 ± 59.61	<0.001
**Demographic characteristics**					
Age in years	55.56 ± 10.15	53.97 ± 9.83	53.89 ± 9.67	52.30 ± 8.61	<0.001
**Sex**					
Men (%)	43.1	44.5	44.5	48.7	0.033
Women (%)	56.9	55.5	55.5	51.3	
**Education (%)**					
< High school	94.7	94.0	93	92.7	0.307
High school or vocational	4.7	5.3	6.3	6.2	
≥College	0.6	0.7	0.7	1.1	
**Behavioral characteristics**					
Physical activity (min/day)	33.22 ± 24.05	34.73 ± 24.78	35.45 ± 22.54	35.78 ± 20.91	0.127
**Alcohol (%)**					
0/week	81.0	81.1	82.6	75.1	<0.001
1–3/week	9.8	11.8	8.6	9.3	
>3/week	8.2	5.9	8.3	14.7	
Unknown	1.0	1.2	0.5	0.9	
**Smoking (%)**					
Never	70.7	69.6	70.9	67.5	0.123
Former	2.4	1.7	1.6	1.9	
Current	24.3	26.8	26.3	27.8	
Unknown	2.6	1.9	1.2	2.8	
Sleep duration in hours/day, mean (SD)	7.22 ± 1.07	7.32 ± 1.01	7.25 ± 1.08	7.45 ± 0.80	<0.001
Television watching in hours/day, mean (SD)	6.37 ± 1.88	3.70 ± 3.14	3.73 ± 2.75	3.64 ± 2.82	<0.001
**Food groups in g/day, mean (SD)**					
Fruits	91.88 ± 113.91	92.66 ± 93.98	136.66 ± 110.86	155.01 ± 82.97	<0.001
Vegetables	298.47 ± 126.80	315.06 ± 122.18	319.25 ± 166.58	368.45 ± 158.25	<0.001
Red meat	93.87 ± 44.34	61.53 ± 46.06	45.97 ± 49.03	41.59 ± 29.34	<0.001
Poultry	33.07 ± 46.69	34.12 ± 26.90	48.44 ± 44.37	94.90 ± 49.98	<0.001
Fish	50.97 ± 39.61	51.87 ± 49.46	78.33 ± 75.02	108.45 ± 68.01	<0.001
Dairy	16.53 ± 50.46	18.36 ± 62.84	20.83 ± 56.99	29.06 ± 106.87	<0.001
Nuts	6.26 ± 12.42	7.17 ± 25.20	8.76 ± 17.03	9.66 ± 13.18	<0.001
Salted vegetables	13.35 ± 17.16	17.14 ± 12.54	21.77 ± 32.26	39.73 ± 40.66	<0.001
**Cardiometabolic markers**					
Body weight in kg, mean (SD)	63.86 ± 10.53	61.14 ± 9.48	60.34 ± 9.15	59.79 ± 9.62	<0.001
BMI, mean (SD)	24.78 ± 3.23	23.45 ± 3.01	23.82 ± 2.89	23.68 ± 2.88	<0.001
Waist circumference in cm, mean (SD)	84.75 ± 9.01	81.41 ± 8.29	80.21 ± 8.19	80.81 ± 7.68	<0.001
Triglycerides in mmol/L, mean (SD)	1.68 ± 1.36	1.55 ± 1.26	1.50 ± 1.03	1.54 ± 1.10	0.001
HDL cholesterol in mmol/L, mean (SD)	1.33 ± 0.33	1.34 ± 0.33	1.36 ± 0.32	1.37 ± 0.31	0.003
LDL cholesterol in mmol/L, mean (SD)	2.79 ± 0.75	2.81 ± 0.73	2.82 ± 0.73	2.87 ± 0.76	0.047
Systolic BP in mm/Hg, mean (SD)	137.83 ± 17.87	136.00 ± 16.68	134.33 ± 14.15	132.03 ± 14.55	<0.001
Diastolic BP in mm/Hg, mean (SD)	87.74 ± 8.15	86.80 ± 8.54	86.47 ± 8.03	85.83 ± 7.54	<0.001
Glucose in mmol/L, mean (SD)	6.39 ± 0.75	5.36 ± 0.95	5.69 ± 1.10	5.57 ± 0.90	<0.001

BP, blood pressure; HDL, high density lipoproteins; LDL, low density lipoproteins; SD, standard deviation.

* P-values were calculated using one-way analysis of variance (ANOVA) for continuous variables and the chi-squared (χ^2^) test for categorical variables to assess overall group differences across quartiles of soy food consumption. No formal *post hoc* pairwise comparisons were conducted.

The prevalence of MetS and its individual components in the study population was as follows: MetS (52.1%), elevated waist circumference (58.3%), elevated triglycerides (35.8%), reduced HDL cholesterol (54.1%), elevated blood pressure (55.9%), and elevated fasting blood glucose (53.4%).

Table 2 presents the crude and adjusted ORs for MetS and its components across quartiles of soy consumption. After adjustment for potential confounders, higher soy consumption was associated with significantly lower odds of MetS and all components except elevated fasting blood glucose (*P* for trend ≤ 0.024). When modeled as a continuous variable, each 25 g/day increase in soy consumption was associated with lower odds of MetS (OR 0.95, 95% CI 0.92–0.98), elevated waist circumference (OR 0.97, 95% CI 0.94–0.99), elevated triglycerides (OR 0.94, 95% CI 0.91–0.96), reduced HDL cholesterol (OR 0.95, 95% CI 0.92–0.97), and elevated blood pressure (OR 0.97, 95% CI 0.94–0.99).

**Table 2:** Odds ratios (ORs) and 95% confidence intervals (CIs) for metabolic syndrome and its components according to soy consumption.

**Outcome**	**Q1 (*n* = 1,294)**	**Q2 (*n* = 1,333)**	**Q3 (*n* = 1,260)**	**Q4 (*n* = 1,220)**	***P*-value for linear trend**	***R* ^2^**	**Each 25 g/day increment (*n* = 5,107)**
**Metabolic syndrome**							
Cases (%)	59.0	50.5	49.4	48.8			52.1
Crude OR (95% CI)	Reference	**0.70 (0.61, 0.81)**	**0.68 (0.56, 0.81)**	**0.66 (0.56, 0.77)**	<0.001	0.02	**0.94 (0.92, 0.97)**
Adjusted OR (95% CI)^a^	Reference	**0.72 (0.63, 0.82)**	**0.70 (0.57, 0.83)**	**0.68 (0.59, 0.80)**	<0.001	0.03	**0.95 (0.92, 0.98)**
**Elevated waist circumference**							
Cases (%)	61.0	57.9	56.8	56.7			58.3
Crude OR (95% CI)	Reference	**0.87 (0.74, 1.00)**	**0.83 (0.69, 0.98)**	**0.82 (0.70, 0.95)**	0.041	0.01	**0.97 (0.94, 1.00)**
Adjusted OR (95% CI)^a^	Reference	**0.86 (0.72, 0.99)**	**0.82 (0.68, 0.97)**	**0.80 (0.69, 0.94)**	0.024	0.02	**0.97 (0.94, 0.99)**
**Elevated triglyceride**							
Cases (%)	40.2	36.5	33.0	31.7			35.8
Crude OR (95% CI)	Reference	**0.86 (0.74, 0.99)**	**0.73 (0.60, 0.87)**	**0.70 (0.60, 0.80)**	<0.001	0.01	**0.94 (0.92, 0.97)**
Adjusted OR (95% CI)^a^	Reference	**0.82 (0.69, 0.96)**	**0.71 (0.58, 0.84)**	**0.68 (0.58, 0.78)**	<0.001	0.02	**0.94 (0.91, 0.96)**
**Reduced HDL-cholesterol**							
Cases (%)	62.8	52.5	53.0	49.8			54.1
Crude OR (95% CI)	Reference	**0.79 (0.68, 0.89)**	**0.72 (0.60, 0.84)**	**0.71 (0.60, 0.82)**	0.001	0.01	**0.94 (0.91, 0.96)**
Adjusted OR (95% CI)^a^	Reference	**0.83 (0.71, 0.94)**	**0.75 (0.63, 0.87)**	**0.73 (0.62, 0.85)**	0.001	0.02	**0.95 (0.92, 0.97)**
**Elevated blood pressure**							
Cases (%)	60.6	56.2	52.0	53.2			55.9
Crude OR (95% CI)	Reference	**0.83 (0.72, 0.95)**	**0.79 (0.65, 0.94)**	**0.74 (0.63, 0.86)**	0.002	0.01	**0.95 (0.93, 0.98)**
Adjusted OR (95% CI)^a^	Reference	**0.89 (0.78, 0.99)**	**0.84 (0.69, 0.98)**	**0.81 (0.69, 0.92)**	0.017	0.01	**0.97 (0.94, 0.99)**
**Elevated fasting blood glucose**							
Cases (%)	53.2	53.4	53.8	53.4			53.4
Crude OR (95% CI)	Reference	1.03 (0.87, 1.16)	1.05 (0.88, 1.22)	1.01 (0.86, 1.18)	0.931	0.01	1.00 (0.97, 1.03)
Adjusted OR (95% CI)^a^	Reference	0.98 (0.82, 1.11)	1.01 (0.84, 1.18)	0.96 (0.81, 1.15)	0.670	0.01	0.99 (0.96, 1.02)

HDL, high density lipoproteins; LDL, low density lipoproteins.

Bold numbers indicate statistical significance (P < 0.05).

Crude and adjusted ORs and 95% CIs were estimated using multivariable logistic regression.

a Models were adjusted for age, sex, education, smoking, alcohol, physical activity, body mass index, sleep duration, television watching, and consumption of red meat, poultry, fish, nuts, salted vegetables, vegetables, and fruits.

Nagelkerke R^2^ values were calculated separately for crude and adjusted models and represent the proportion of variance in each outcome explained by soy consumption alone (crude models) or by soy consumption and the included covariates (adjusted models).

Table 3 shows sex-stratified associations. Among men, higher soy consumption was associated with lower odds of MetS, elevated triglycerides, and reduced HDL cholesterol compared to the lowest quartile, although linear trends were not statistically significant (*P* for trend ≥0.061). In contrast, a clear dose-response relationship was observed among women, with higher soy consumption associated with progressively lower odds of MetS and all components except elevated fasting blood glucose (*P* for trend ≤ 0.003). For each 25 g/day increase in soy consumption, women had lower odds of MetS (OR 0.92, 95% CI 0.90–0.95), elevated waist circumference (OR 0.96, 95% CI 0.93–0.99), elevated triglycerides (OR 0.92, 95% CI 0.89–0.94), reduced HDL cholesterol (OR 0.91, 95% CI 0.89–0.93), and elevated blood pressure (OR 0.96, 95% CI 0.94–0.98).

**Table 3:** Sex-specific adjusted odds ratios (ORs) and 95% confidence intervals (CIs) for metabolic syndrome and its components according to soy consumption.

**Outcome**	**Q1**	**Q2**	**Q3**	**Q4**	***P*-value for linear trend**	***R^2^***	**Each 25 g/day increment**
**Men (*****n*** = **2,284)**							
Metabolic syndrome	Reference	0.86 (0.75, 1.01)	**0.84 (0.71, 0.99)**	**0.83 (0.71, 0.98)**	0.097	0.01	0.97 (0.94, 1.00)
Elevated waist circumference	Reference	0.89 (0.74, 1.12)	0.88 (0.77, 1.02)	0.84 (0.73, 1.01)	0.055	0.00	0.97 (0.95, 1.00)
Elevated triglyceride	Reference	0.87 (0.77, 1.02)	**0.85 (0.72, 0.99)**	**0.83 (0.71, 0.96)**	0.061	0.01	0.97 (0.95, 1.00)
Reduced HDL-cholesterol	Reference	0.86 (0.76, 1.03)	**0.85 (0.74, 0.96)**	**0.84 (0.72, 0.97)**	0.077	0.00	0.98 (0.95, 1.00)
Elevated blood pressure	Reference	1.08 (0.91, 1.29)	0.88 (0.76, 1.13)	0.87 (0.76, 1.01)	0.100	0.01	0.97 (0.94, 1.00)
Elevated fasting blood glucose	Reference	1.22 (0.95, 1.97)	1.03 (0.51, 2.02)	1.11 (0.95, 1.32)	0.348	0.00	1.01 (0.99, 1.04)
**Women (*****n*** = **2,823)**							
Metabolic syndrome	Reference	**0.68 (0.59, 0.78)**	**0.64 (0.54, 0.74)**	**0.60 (0.52, 0.68)**	<0.001	0.04	**0.92 (0.90, 0.95)**
Elevated waist circumference	Reference	**0.80 (0.68, 0.93)**	**0.77 (0.67, 0.88)**	**0.76 (0.65, 0.87)**	0.003	0.03	**0.96 (0.93, 0.99)**
Elevated triglyceride	Reference	**0.76 (0.65, 0.88)**	**0.62 (0.54, 0.71)**	**0.59 (0.51, 0.68)**	<0.001	0.03	**0.92 (0.89, 0.94)**
Reduced HDL-cholesterol	Reference	**0.77 (0.66, 0.89)**	**0.68 (0.59, 0.78)**	**0.58 (0.50, 0.66)**	0.001	0.03	**0.91 (0.89, 0.93)**
Elevated blood pressure	Reference	**0.75 (0.64, 0.87)**	**0.82 (0.70, 0.95)**	**0.75 (0.66, 0.85)**	0.001	0.02	**0.96 (0.94, 0.98)**
Elevated fasting blood glucose	Reference	0.77 (0.52, 1.03)	0.97 (0.75, 1.19)	0.92 (0.80, 1.05)	0.505	0.00	0.99 (0.97, 1.01)

HDL, high-density lipoproteins; LDL, low-density lipoproteins.

Bold numbers indicate statistical significance (P < 0.05).

Adjusted ORs and 95% CIs were estimated using multivariable logistic regression.

Models were adjusted for age, education, smoking, alcohol, physical activity, body mass index, sleep duration, television watching, and consumption of red meat, poultry, fish, nuts, salted vegetables, vegetables, and fruits.

Model fit was assessed using Nagelkerke R^2^, which quantifies the proportion of variation in the outcome explained by the logistic regression model.

Nagelkerke R^2^ values represent the proportion of variance in each outcome explained by soy consumption and the included covariates.

Table 4 presents results stratified by menopausal status. In both premenopausal and postmenopausal women, higher soy consumption was consistently associated with lower odds of MetS and all components except elevated fasting blood glucose. Statistically significant linear trends were observed across soy consumption quartiles in both groups (*P* for trend ≤ 0.009 in premenopausal women; ≤ 0.001 in postmenopausal women). For each 25 g/day increase in soy consumption, premenopausal women had lower odds of MetS (OR 0.93, 95% CI 0.90–0.96), elevated waist circumference (OR 0.96, 95% CI 0.94–0.99), elevated triglycerides (OR 0.92, 95% CI 0.89–0.94), reduced HDL cholesterol (OR 0.92, 95% CI 0.89–0.94), and elevated blood pressure (OR 0.97, 95% CI 0.95–0.99). The corresponding ORs in postmenopausal women were 0.91 (95% CI 0.89–0.94), 0.95 (95% CI 0.93–0.98), 0.91 (95% CI 0.88–0.93), 0.90 (95% CI 0.87–0.92), and 0.95 (95% CI 0.93–0.98), respectively.

**Table 4:** Adjusted odds ratios (ORs) and 95% confidence intervals (CIs) for metabolic syndrome and its components according to soy consumption in premenopausal and postmenopausal women.

**Outcome**	**Q1**	**Q2**	**Q3**	**Q4**	***P*-value for linear trend**	***R^2^***	**Each 25 g/day increment**
**Premenopausal women (*****n*** = **758)**							
Metabolic syndrome	Reference	**0.70 (0.61, 0.80)**	**0.67 (0.57, 0.77)**	**0.63 (0.55, 0.72)**	<0.001	0.04	**0.93 (0.90, 0.96)**
Elevated waist circumference	Reference	**0.84 (0.71, 0.97)**	**0.80 (0.69, 0.91)**	**0.78 (0.67, 0.88)**	0.002	0.02	**0.96 (0.94, 0.99)**
Elevated triglyceride	Reference	**0.78 (0.67, 0.90)**	**0.65 (0.56, 0.74)**	**0.61 (0.53, 0.70)**	<0.001	0.03	**0.92 (0.89, 0.94)**
Reduced HDL-cholesterol	Reference	**0.79 (0.68, 0.91)**	**0.71 (0.62, 0.81)**	**0.61 (0.52, 0.69)**	<0.001	0.03	**0.92 (0.89, 0.94)**
Elevated blood pressure	Reference	**0.77 (0.62, 0.83)**	**0.85 (0.73, 0.98)**	**0.79 (0.70, 0.90)**	0.009	0.02	**0.97 (0.95, 0.99)**
Elevated fasting blood glucose	Reference	0.83 (0.57, 1.09)	1.02 (0.81, 1.23)	0.96 (0.84, 1.08)	0.822	0.00	1.00 (0.98, 1.02)
**Postmenopausal women (*****n*** = **2,065)**							
Metabolic syndrome	Reference	**0.66 (0.55, 0.76)**	**0.62 (0.51, 0.72)**	**0.58 (0.50, 0.65)**	<0.001	0.04	**0.91 (0.89, 0.94)**
Elevated waist circumference	Reference	**0.78 (0.65, 0.90)**	**0.75 (0.65, 0.86)**	**0.73 (0.62, 0.84)**	0.001	0.02	**0.95 (0.93, 0.98)**
Elevated triglyceride	Reference	**0.72 (0.62, 0.83)**	**0.60 (0.50, 0.70)**	**0.57 (0.49, 0.65)**	<0.001	0.03	**0.91 (0.88, 0.93)**
Reduced HDL-cholesterol	Reference	**0.73 (0.63, 0.84)**	**0.64 (0.55, 0.75)**	**0.53 (0.45, 0.62)**	<0.001	0.03	**0.90 (0.87, 0.92)**
Elevated blood pressure	Reference	**0.73 (0.63, 0.84)**	**0.80 (0.68, 0.93)**	**0.71 (0.62, 0.81)**	<0.001	0.02	**0.95 (0.93, 0.98)**
Elevated fasting blood glucose	Reference	0.74 (0.47, 1.01)	0.93 (0.70, 1.15)	0.89 (0.75, 1.02)	0.347	0.00	0.99 (0.96, 1.01)

HDL, high-density lipoproteins; LDL, low-density lipoproteins.

Bold numbers indicate statistical significance (P < 0.05).

Adjusted ORs and 95% CIs were estimated using multivariable logistic regression.

Models were adjusted for age, education, smoking, alcohol, physical activity, body mass index, sleep duration, television watching, and consumption of red meat, poultry, fish, nuts, salted vegetables, vegetables, and fruits.

Nagelkerke R^2^ values represent the proportion of variance in each outcome explained by soy consumption and the included covariates.

## Discussion

In the present cross-sectional study of 5,107 Chinese adults, soy consumption was inversely associated with the odds of MetS and all of its components except elevated fasting blood glucose. These associations appeared to be stronger in women than in men. Among women, menopausal status did not appear to modify the observed associations.

Our finding that increased soy consumption was associated with lower odds of having MetS is generally in line with the reduced incidence of MetS reported with higher soy protein intake in a prospective cohort study (13) and the reduced prevalence of MetS observed with moderate soy consumption in a cross-sectional study (18). However, such beneficial associations were not evident in other studies (14–17). The reasons for these discrepancies remain unclear. In most studies that found a null association (15–17), soy consumption was examined as one of many potential factors, rather than the primary exposure of interest. Consequently, the variables controlled for in multivariable models may not have been optimal for assessing the soy–MetS association. Notably, these studies did not adjust for dietary factors, which may have contributed to differing results.

There is some indication that the relationship between soy or soy protein consumption and MetS could differ by sex. A prospective cohort study (13) found an inverse association between soy protein intake and MetS incidence in women but not in men. Another study (14) suggested a trend toward increased risk in men and decreased risk in women, although these trends were not statistically significant. Consistent with these findings, we observed that the inverse association between soy consumption and MetS was stronger in women than in men. Soy consumption was also more consistently associated with lower odds of elevated waist circumference, elevated triglycerides, reduced HDL cholesterol, and elevated blood pressure in women. A growing body of evidence supports potential beneficial effects of soy or isoflavones on individual MetS components (7–12), although this evidence largely derives from trials in women, with limited data available in men. Thus, current evidence remains insufficient to determine whether the association between soy consumption and MetS differs by sex.

The biological mechanisms underlying potential sex differences remain unclear. Soy foods are rich in phytoestrogens (isoflavones), which can mimic the structure and function of endogenous estrogens. This has prompted investigations into possible hormonal effects in men through reduced testosterone concentrations (28, 29) or changes in sex hormone-binding globulin (SHBG), which modulates free testosterone levels (30–34). Since lower testosterone concentrations may be associated with increased risk of MetS in men but reduced risk in women (35), hormonal modulation could partly explain sex-specific effects. However, meta-analyses suggest that soy or isoflavone supplementation does not significantly alter testosterone or SHBG concentrations in either men (28, 29) or women (36), and the relevance of these mechanisms remains uncertain. We did not collect biochemical data on sex hormones such as testosterone, estradiol, or SHBG in the present study. As such, we were unable to examine whether hormonal pathways may have contributed to the sex- or menopause-specific associations we observed.

Because isoflavones can bind estrogen receptors and exert weak estrogenic effects (37), their health impacts may differ depending on endogenous estrogen levels. Premenopausal women have higher estradiol levels than postmenopausal women, raising the possibility that isoflavones may interfere with endogenous estrogen activity in premenopausal women but act as weak estrogens in postmenopausal women. However, to our knowledge, limited data are available on how menopausal status may influence these associations. In the present study, similar inverse trends were observed between soy consumption and MetS and its components (excluding fasting blood glucose) among both premenopausal and postmenopausal women, suggesting that menopausal status did not modify these associations. A meta-analysis of clinical trials (31) also found no significant effect of soy or isoflavone supplementation on estradiol concentrations in either group, which may explain the absence of effect modification by menopausal status.

Several caveats must be considered. First, the cross-sectional design precludes causal inference, as exposure and outcome were assessed simultaneously. Second, soy consumption was assessed using an FFQ, which is inherently subject to recall and reporting bias. Participants may overreport foods perceived as healthy, such as soy, and underreport foods perceived as unhealthy. Additionally, soy consumption was assessed using a single FFQ item that did not differentiate specific soy products (e.g., tofu, soy milk, soybean paste, soybeans). Because nutrient profiles vary substantially among soy items (e.g., tofu, soy milk, soybean paste), the inability to distinguish these products may have limited the ability to detect product-specific associations. Third, despite adjustment for major covariates known to be associated with MetS (5), residual confounding cannot be ruled out, especially in the absence of energy or nutrient data. Fourth, we used the JIS criteria to define MetS, which are widely applied and include Asian-specific cut-offs (17, 23–26). However, prevalence estimates may differ under alternative definitions. For example, the CDS criteria use stricter thresholds, including a higher cut-off for waist circumference in women (≥85 vs. ≥80 cm in JIS) and fasting glucose (≥6.1 vs. ≥5.6 mmol/L), and do not use sex-specific HDL cholesterol thresholds. These definitional differences contribute to higher prevalence estimates when using the JIS criteria. However, the JIS definition has demonstrated stronger predictive value for MetS in Chinese populations (25, 26), supporting its use in this study. Finally, the study population was drawn from Suzhou Industrial Park, located in the Jiangnan region of southeastern China. This region is known for its distinctive “Jiangnan diet,” characterized by high consumption of seasonal vegetables, legumes (particularly soy), and freshwater fish; moderate consumption of whole-grain rice and red meat; and low consumption of salt-preserved foods (38). Although the study was conducted in an urban and economically developed area, participants predominantly reported modest household incomes (2,000–4,000 RMB/month) and low educational attainment (over 90% had high school education or below). Therefore, the generalizability of our findings to the broader Chinese populations—particularly those with differing dietary habits or sociodemographic profiles—may be limited.

## Conclusions

In this cross-sectional study of Chinese adults, higher soy consumption was associated with lower likelihood of MetS and most of its components, except elevated fasting blood glucose, particularly among women. While these findings suggest a potential role of soy in supporting metabolic health, public health recommendations should await confirmation from longitudinal and intervention studies. Future research is needed to replicate these associations in diverse populations, differentiate the effects of specific soy products, and assess whether associations vary by sex or menopausal status. Until stronger evidence becomes available, soy may be considered part of a balanced dietary pattern rather than a targeted preventive strategy for MetS.

## Data Availability

The raw data supporting the conclusions of this article will be made available by the authors, without undue reservation.
